# When and How Head Nurses’ Ethical Leadership Shapes Nurses’ Creative Patient Service: The Roles of Ethical Generative AI Usage and AI Compatibility

**DOI:** 10.1155/jonm/5559768

**Published:** 2026-09-30

**Authors:** Yi Liu

**Affiliations:** ^1^ School of Economics and Management Shijiazhuang University Shijiazhuang 050035 Hebei China sjzc.edu.cn

**Keywords:** AI compatibility, creative patient service, ethical leadership, ethical usage, GenAI

## Abstract

**Aims:**

To investigate the impact of head nurses’ ethical leadership on nurses’ creative patient service, specifically examining the mediating role of ethical generative artificial intelligence (GenAI) usage and the moderating influence of perceived artificial intelligence (AI) compatibility.

**Background:**

The rapid integration of GenAI tools into clinical nursing offers opportunities for innovation. While ethical leadership is known to influence employee behaviour, its role in guiding ethical technology use to drive creative patient care remains under‐explored in the digitalised healthcare environment.

**Methods:**

A multisource, dyadic study was conducted across 12 tertiary hospitals in four major Chinese cities. Using convenience sampling, we recruited 64 head nurses and 592 matched staff nurses. The target sample size was determined a priori through power analysis. Staff nurses rated their head nurse’s ethical leadership, their own ethical GenAI usage and their perceived AI compatibility. Head nurses rated each staff nurse’s creative patient service. Hypotheses were tested using path analysis in Mplus 8.3.

**Results:**

Ethical leadership from head nurses was positively related to nurses’ creative patient service. This relationship was partially mediated by nurses’ GenAI ethical usage. AI compatibility acted as a moderator; the indirect positive effect of ethical leadership on creative service via GenAI usage was stronger for nurses with higher levels of AI compatibility.

**Conclusions:**

Head nurses play a central role in the AI era by modelling ethical standards that encourage the responsible use of GenAI. When nurses use these tools ethically and possess high technological compatibility, they are better equipped to provide creative solutions in patient care.

**Implications for Nursing Management:**

Nursing administrators should prioritise ethical leadership training to guide the responsible adoption of AI technologies. Management should not only focus on the technical implementation of GenAI but also build a culture of ethical usage and assess individual AI compatibility. Tailored support for nurses with lower AI compatibility may be necessary to ensure equitable innovation in patient service across the workforce.

## 1. Introduction

The rapid integration of artificial intelligence (AI), including generative AI (GenAI) tools, into healthcare delivery is reshaping nursing practice by offering opportunities for more efficient, data‐informed and personalised patient care [1]. In clinical nursing settings, GenAI supports tasks such as synthesising patient information, generating care ideas and streamlining documentation, which can ease administrative burdens amid rising workloads, high patient acuity, staffing shortages and turnover challenges that are particularly acute in tertiary hospitals in China and globally [2]. Successful AI adoption, however, is fundamentally a socio‐technical endeavour, requiring alignment of technological capabilities with organisational structures, human factors and ethical safeguards [3]. Nursing leaders, particularly head nurses, are central to this process, as they guide teams through transitions by promoting AI literacy, addressing rational resistance stemming from concerns over workflow disruption, professional autonomy, patient safety and algorithmic bias, and ensuring integration remains context‐specific, ethically grounded and human‐centred [4].

Despite growing recognition of leadership’s role in enabling ethical and effective AI engagement, significant gaps remain in the literature. While studies have explored nurses’ perceptions, attitudes and readiness towards AI, as well as general leadership influences on technology adoption [5–7], limited attention has been given to how ethical leadership from head nurses specifically supports nurses’ ethical GenAI usage, encompassing responsible practices such as privacy protection, output verification, transparency and professional judgement [8]. In addition, the pathway through which ethical leadership translates into enhanced creative patient service (innovative, resourceful approaches to meeting unique patient needs and improving satisfaction) is under‐explored [9]. Key moderators, such as nurses’ perceived AI compatibility (alignment with existing technologies, workflows, unit culture and professional values), have received scant empirical examination in determining the conditions under which leadership effects are amplified. These gaps are especially relevant in high‐pressure environments like Chinese tertiary hospitals, where systemic demands heighten the need for evidence‐based strategies that ethically harness GenAI to mitigate workforce challenges and promote innovation [10].

Conservation of resources (COR) theory [11] holds that individuals strive to acquire, retain and protect valued resources while limiting losses in stressful settings. Drawing on this view, the present multi‐source study examines the relationships among head nurses’ ethical leadership (rated by staff nurses), nurses’ ethical GenAI usage and nurses’ creative patient service (rated by head nurses), with perceived AI compatibility as a moderator. Ethical leadership serves as a critical external resource that models integrity, fairness and value‐based decision‐making, thereby helping nurses conserve and gain psychological and professional resources (for example, trust, ethical confidence and motivation) to engage responsibly with GenAI. In turn, ethical GenAI usage acts as a resource investment mechanism, enabling nurses to use GenAI outputs creatively without depleting personal resources or risking ethical breaches.

This study advances understanding of nursing leadership in AI integration by showing that ethical leadership indirectly enhances creative patient service through ethical GenAI usage, with the effect stronger under high AI compatibility, positioning compatibility as a boundary condition that amplifies resource gain spirals. By integrating COR theory within a socio‐technical framework [12], the findings provide evidence‐based insights for nursing leaders to prioritise ethical role modelling alongside targeted initiatives that build AI compatibility, thereby enabling resilient, innovative and equitable GenAI adoption amid workforce pressures [13]. These contributions align with calls for adaptive leadership strategies that address ethical considerations, organisational readiness and workforce challenges to support sustainable AI‐driven nursing practice in demanding clinical settings [14]. Figure 1 presents the conceptual framework of the study.

**FIGURE 1:**
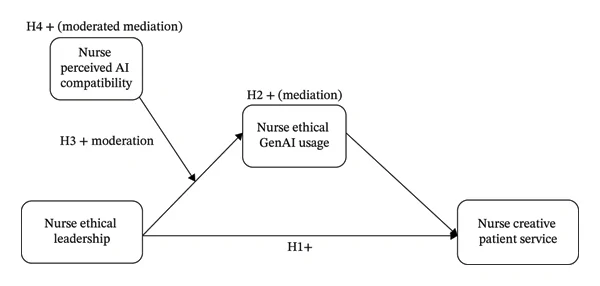
Conceptual framework. *Source*: Author(s) own work.

## 2. Theory and Hypotheses

### 2.1. COR Theory

COR theory [11, 15] posits that individuals are motivated to acquire, retain, protect and foster valued resources like objects, personal characteristics, conditions and energies that help meet goals and manage demands. The theory emphasises the primacy of resource loss, where actual or threatened losses are more psychologically impactful than equivalent gains, often triggering defensive behaviours to conserve resources. It also highlights resource investment to prevent future losses or generate gains, leading to loss spirals (accelerating depletion) or gain spirals (accumulating further resources), especially in high‐stress environments like healthcare [15].

Applying COR theory to this study, ethical leadership from head nurses acts as a vital external resource in resource‐scarce Chinese tertiary hospital settings, modelling integrity and providing ethical guidance to help nurses conserve psychological resources (e.g., trust, ethical confidence) and invest them in responsible GenAI usage. Ethical GenAI usage becomes a resource investment mechanism, enabling creative patient service while protecting against losses from misuse. AI compatibility serves as a key boundary condition: high compatibility lowers perceived threats, strengthens resource gain spirals from leadership to ethical usage and amplifies creative outcomes; low compatibility heightens loss risks and weakens the pathway. Thus, COR theory explains how ethical leadership initiates protective and gain‐oriented processes to ethically integrate GenAI and enhance innovative nursing practice amid workforce pressures.

### 2.2. Head Nurse Ethical Leadership and Creative Patient Service

Ethical leadership in nursing, characterised by integrity, fairness, role modelling and emphasis on ethical standards, has been conceptually linked to enhanced employee performance and innovation in healthcare settings [16]. Head nurses who exhibit ethical leadership create a supportive environment where staff nurses feel empowered to explore novel approaches to patient care, such as improvising solutions for unique needs or viewing challenges from fresh perspectives, thereby promoting creative patient service [17]. Empirical evidence supports this association; for instance, studies in nursing management have shown that ethical leaders improve job satisfaction and creativity by encouraging value‐aligned behaviours and reducing ethical dilemmas in high‐pressure clinical contexts [18]. Recent evidence from a large sample of Chinese nurses similarly shows that ethical leadership promotes proactive behaviours such as voice through psychological mechanisms, reinforcing its role as a driver of constructive nurse conduct [19]. In Chinese tertiary hospitals, where nurses face intense workloads and resource constraints, ethical leadership likely motivates nurses to channel their efforts into innovative, patient‐centred service delivery to achieve better outcomes [20].

Drawing on COR theory, ethical leadership serves as a key external resource that helps nurses conserve and build personal resources like ethical confidence and motivation in demanding environments. By modelling ethical decision‐making and prioritising the ‘right thing to do’, head nurses mitigate resource losses associated with moral distress or uncertainty, enabling nurses to invest surplus resources in creative endeavours. This initiates gain spirals, where protected resources (e.g., trust in leadership) translate into proactive, innovative behaviours such as generating creative ideas for patient satisfaction, ultimately enhancing creative patient service [21].

H1: Head nurse ethical leadership is positively related to nurse creative patient service.

### 2.3. Nurse Ethical GenAI Usage as a Mediator

Conceptually, ethical leadership influences creative outcomes indirectly by promoting responsible technology adoption, as leaders who discuss ethics and set examples encourage nurses to use GenAI tools in transparent, verified and judgement‐based ways that align with professional standards [22]. This ethical usage, in turn, equips nurses with reliable GenAI outputs for innovative applications, such as generating novel care ideas or efficient methods, thereby bridging leadership to creative patient service. Empirical studies in healthcare and technology adoption contexts have demonstrated similar mediation effects; for example, ethical leadership has been found to mediate technology integration and performance through ethical behaviours, with GenAI‐specific research showing that responsible usage enhances creativity in clinical problem‐solving [23–25].

Drawing on COR theory, ethical leadership provides resources like ethical guidance and trust, which nurses invest in ethical GenAI usage to avoid losses from misuse (e.g., privacy breaches or autonomy erosion). This investment creates gain spirals, where ethical usage conserves cognitive resources and generates new ones (e.g., validated insights), allowing nurses to apply GenAI creatively without depletion, thus mediating the path to enhanced patient service [11, 21].

H2: Nurse ethical GenAI usage mediates the relationship between head nurse ethical leadership and nurse creative patient service.

### 2.4. Nurse AI Compatibility as a Moderator

AI compatibility, reflecting the perceived fit of GenAI with existing workflows, technologies, culture and values, conceptually strengthens leadership effects by reducing barriers to adoption, making ethical guidance more actionable and relevant in aligned contexts [26]. When compatibility is high, nurses are more receptive to ethical leadership’s influence, leading to greater ethical GenAI usage; conversely, low compatibility may hinder this translation due to perceived mismatches. Empirical evidence from technology adoption literature, including AI in healthcare, supports this moderation; studies show that compatibility amplifies leadership impacts on ethical technology behaviours, particularly in nursing where contextual fit influences tool engagement [27, 28].

Through the lens of COR theory, high AI compatibility acts as a facilitating condition that minimises perceived resource threats (e.g., workflow disruption), enabling nurses to more effectively draw on ethical leadership resources for investment in ethical GenAI usage and building stronger gain spirals. In contrast, low compatibility exacerbates loss risks, weakening the leadership‐usage link by forcing resource conservation over investment, thus moderating the pathway in resource‐demanding clinical settings [21].

H3: Nurse AI compatibility moderates the relationship between head nurse ethical leadership and nurse ethical GenAI usage, such that the relationship is stronger (vs. weaker) when AI compatibility is high (vs. low).

Conceptually, the indirect pathway from ethical leadership to creative patient service via ethical GenAI usage is expected to be conditional on the degree to which nurses perceive GenAI as compatible with their existing workflows, technologies, unit culture and professional values. When compatibility is high, ethical leadership’s influence on ethical GenAI usage is amplified because nurses experience less disruption, greater ease of integration and stronger alignment with their professional identity, allowing them to more effectively translate ethical usage into innovative, resourceful patient care solutions. Empirical evidence from technology adoption and leadership literature supports this conditional indirect effect; studies have shown that technology compatibility strengthens mediated pathways in innovation and performance models, particularly in service‐oriented and healthcare contexts where contextual fit moderates the impact of leadership on technology‐enabled outcomes [29–31].

From the perspective of COR theory, high AI compatibility serves as a facilitating resource condition that reduces perceived threats of resource loss (e.g., workflow disruption, increased cognitive load or ethical uncertainty) and enhances the efficiency of resource investment from ethical leadership. When compatibility is high, nurses can more readily convert the external resource of ethical leadership into ethical GenAI usage, creating stronger gain spirals that lead to greater creative patient service. In contrast, low compatibility heightens loss risks, forcing resource conservation rather than investment, thereby weakening the mediated pathway. Thus, COR theory predicts that the indirect effect of ethical leadership on creative patient service through ethical GenAI usage will be stronger at higher levels of AI compatibility.

H4: Nurse AI compatibility moderates the indirect relationship between head nurse ethical leadership and nurse creative patient service through ethical GenAI usage, such that the indirect effect is stronger (vs. weaker) when AI compatibility is high (vs. low).

## 3. Methods

### 3.1. Context

This study was conducted in tertiary (Class III) hospitals in Beijing, Shanghai, Guangzhou and Chengdu, China’s leading metropolitan centres. These large‐scale, university‐affiliated institutions represent the highest level of the national healthcare system, providing advanced specialised care, medical education and research while managing high patient volumes, complex cases and an ageing population with rising chronic disease burden [32]. Nursing in these settings is characterised by intense workloads, suboptimal nurse‐to‐patient ratios, persistent staffing shortages, high turnover and increasing administrative demands, creating a high‐stress, resource‐constrained environment [33]. Amid these pressures, GenAI tools have been rapidly adopted in major Chinese tertiary hospitals since 2025 to support clinical documentation, decision‐making, information synthesis and innovative care planning [34]. However, successful integration remains a socio‐technical challenge, heavily dependent on ethical leadership, workforce readiness, perceived tool compatibility and mitigation of concerns related to patient safety, professional autonomy and algorithmic limitations [35]. This high‐pressure context makes these hospitals an ideal setting to examine how head nurses’ ethical leadership can promote ethical GenAI usage and unlock creative patient service among frontline clinical nurses [36].

### 3.2. Ethical Considerations

This study was conducted in accordance with the Declaration of Helsinki. Ethical approval was obtained from the Institutional Review Board of Shijiazhuang University (Approval No: SJZU‐2025‐0108) on November 05, 2025. Participation was entirely voluntary, and written informed consent was secured from all staff nurses and head nurses prior to data collection. To ensure confidentiality and comply with data privacy protocols, all dyadic data were anonymised using unique matching codes that linked supervisor–subordinate responses without revealing personal identities. Participants were informed of their right to withdraw from the study at any stage without consequence.

### 3.3. Sample and Data Collection

An a priori power analysis was conducted to determine the minimum sample size needed to detect the hypothesised effects. Using the inverse square root method recommended for path models [37], with a minimum detectable path coefficient of 0.2, a significance level of 0.05 and a statistical power of 0.80, the minimum required sample was approximately 160 respondents. This was cross‐checked in G∗Power 3.1 [38] for a linear multiple regression model with three predictors, assuming a medium effect size (*f* ^2^ = 0.15), *α* = 0.05, and a power of 0.80, which returned a minimum of approximately 77 respondents. The achieved sample of 592 staff nurses exceeds both thresholds, indicating that the study was adequately powered to test the proposed relationships.

This study employed a multi‐source, cross‐sectional design to collect dyadic data from head nurses and their staff nurses across 12 tertiary hospitals in four major Chinese cities: Beijing, Shanghai, Guangzhou and Chengdu. The multi‐source design, in which predictor and outcome variables were rated by different sources, was used to reduce the possibility of common method variance [39]. The 12 hospitals were purposively selected based on their integration of GenAI tools into their clinical and patient care systems. This geographic distribution ensured representation across China’s eastern, southern and southwestern regions.

Within the selected hospitals, participants were recruited through convenience sampling. Eligible staff nurses were registered nurses with at least one year of clinical experience who had used GenAI tools in patient care during the preceding six months and who worked under a head nurse willing to participate. Head nurses were eligible if they directly supervised the participating staff nurses. Nurses on extended leave, agency or temporary staff, and those without prior GenAI exposure were excluded. Responses that were incomplete or could not be matched to a supervisor–subordinate dyad were also removed prior to analysis.

With support from each hospital’s nursing department, eligible staff nurses and their head nurses were invited to take part and provided with a secure online survey link. Of the 950 staff nurses invited, 592 returned complete and matched responses, yielding a response rate of 62.32%. In total, 64 head nurses each rated a mean of approximately nine of their direct‐report staff nurses.

Data collection followed a multi‐source approach to minimise common method bias: staff nurses (*n* = 592) rated their respective head nurses’ ethical leadership, their own ethical GenAI usage and their perceived AI compatibility. Head nurses (*n* = 64) rated the creative patient service behaviours of their direct‐report staff nurses. Surveys were administered in Mandarin using a back‐translation procedure [40] through a secure online platform. Participation was voluntary and anonymous, and confidentiality was maintained through unique matching codes that linked supervisor–subordinate responses without revealing identities. Sociodemographic characteristics of the staff nurses and head nurses are summarised in Table 1.

**TABLE 1:** Sociodemographic characteristics of the staff nurse sample (*n* = 592).

Characteristic	Male (*n* = 213) *n* (%)	Female (*n* = 379) *n* (%)	Total (*n* = 592) *n* (%)
Gender	213 (36.0)	379 (64.0)	592 (100.0)
Age (years)			
30 or younger	17 (8.0)	21 (5.5)	38 (6.4)
31–40	110 (51.6)	195 (51.5)	305 (51.5)
41–50	81 (38.0)	145 (38.3)	226 (38.2)
Older than 50	5 (2.3)	18 (4.7)	23 (3.9)
M (SD)	38.19 (6.39)	38.89 (6.44)	38.64 (6.43)
Range	24 to 55	19 to 56	19 to 56
Clinical experience			
	11 (5.2)	29 (7.7)	40 (6.8)
	21 (9.9)	19 (5.0)	40 (6.8)
	22 (10.3)	59 (15.6)	81 (13.7)
	63 (29.6)	108 (28.5)	171 (28.9)
	96 (45.1)	164 (43.3)	260 (43.9)
M (SD)	4.00 (1.19)	3.95 (1.22)	3.96 (1.21)

*Note:* Source: Prepared by the authors based on results from the analysis.

### 3.4. Measures

All constructs were measured using established multi‐item scales adapted to the nursing and GenAI context in Chinese tertiary hospitals. Adaptation involved minor wording changes for clinical relevance while retaining the original item count and response structure. All items were rated on a 5‐point Likert‐type scale (1 = strongly disagree to 5 = strongly agree). For each scale, a higher mean score reflects a higher level of the construct. The original English scales were translated into Mandarin and back‐translated by two independent bilingual experts to ensure conceptual equivalence [40]. Psychometric properties for the current sample are reported in full in the Assessment of the Measurement Model subsection and Table 2; original‐study reliabilities are noted below for each instrument.

**TABLE 2:** Validity and reliability.

Construct/item	FL	AVE	CR	*α*
AI Compatibility (AIC)		0.658	0.884	0.843
AIC1	0.776			
AIC2	0.828			
AIC3	0.907			
AIC4	0.723			
Creative Patient Service (CPS)		0.666	0.933	0.916
CPS1	0.742			
CPS2	0.800			
CPS3	0.761			
CPS4	0.822			
CPS5	0.802			
CPS6	0.885			
CPS7	0.889			
Ethical GenAI Usage (EGU)		0.609	0.903	0.872
EGU1	0.760			
EGU2	0.786			
EGU3	0.796			
EGU4	0.767			
EGU5	0.763			
EGU6	0.809			
Ethical Leadership (EL)		0.618	0.942	0.931
EL1	0.734			
EL2	0.818			
EL3	0.710			
EL4	0.766			
EL5	0.831			
EL6	0.769			
EL7	0.829			
EL8	0.817			
EL9	0.753			
EL10	0.825			

*Note:* Source: Prepared by the authors based on results from the analysis.

Abbreviations: AVE, average variance extracted; CR, composite reliability; FL, factor loading.

*Ethical leadership* was measured using the 10‐item Ethical Leadership Scale developed by Brown, Treviño and Harrison (2005) [41]. Staff nurses rated their head nurse on behaviours reflecting integrity, fairness and ethical role modelling (sample item: ‘My head nurse listens to the concerns and suggestions of the nursing staff’). Item scores were averaged to form an overall ethical leadership score, with higher scores indicating stronger perceived ethical leadership. The original scale demonstrated sound construct validity and reliability (*α* = 0.92) in Brown et al. (2005) [41]. In the current sample, the scale showed high reliability (*α* = 0.931, CR = 0.942) and convergent validity (AVE = 0.618).

*Ethical GenAI usage* was measured using a 6‐item scale developed for this study, informed by the technology usage and attitude framework of Rosen et al. (2013) [42] and adapted to the ethical dimensions of GenAI use in nursing practice, including data‐privacy protection, output verification, transparency and professional judgement (sample item: ‘I ensure my use of GenAI in daily work adheres to patient data privacy protocols’). Item scores were averaged, with higher scores indicating more ethical GenAI usage. Content validity was supported through a review of the items by a panel of five nursing faculty and clinical experts for relevance and clarity. In the current sample, the scale showed high reliability (*α* = 0.872, CR = 0.903) and convergent validity (AVE = 0.609).

Head nurses rated each staff nurse’s *creative patient service* using a 7‐item scale adapted from the salesperson creative performance measure of Wang and Netemeyer (2004) [43]. Items assess innovative and resourceful approaches to meeting patient needs (sample item: ‘This nurse presents services or offerings to patients in innovative and engaging ways’). Item scores were averaged, with higher scores indicating greater creative patient service. The original scale demonstrated strong validity and reliability (*α* = 0.91) in Wang and Netemeyer (2004) [43]. In the current sample, reliability was high (*α* = 0.916, CR = 0.933) with convergent validity (AVE = 0.666).

*Perceived AI compatibility* was measured using a 4‐item scale adapted from Shafique et al. (2026) [44]. Items assess the perceived fit of GenAI with existing infrastructure, workflows and professional values (sample item: ‘GenAI is compatible with the current digital infrastructure used in our ward/hospital’). Item scores were averaged, with higher scores indicating greater perceived compatibility. The original scale reported acceptable reliability (*α* = 0.957) [44]. In the current sample, reliability was acceptable (*α* = 0.843, CR = 0.884) with convergent validity (AVE = 0.658).

### 3.5. Data Analysis

Table 3 presents the descriptive statistics (means and standard deviations) and correlations among the study variables. All core constructs (ethical leadership, ethical GenAI usage, creative patient service and AI compatibility) were measured on a 5‐point Likert‐type scale, with means ranging from 3.95 to 4.19. Correlations among the constructs were low to moderate, indicating no risk of multicollinearity and supporting their empirical distinctiveness. Most demographic variables (gender, age and experience) showed weak correlations with the study constructs; where associations reached significance, they were small in magnitude. To assess their influence, the structural model was estimated both with and without age, gender and experience as control variables. The sign, direction and significance of all hypothesised paths remained unchanged across both specifications. For parsimony, we therefore report the results without controls.

**TABLE 3:** Descriptive statistics and correlations.

Construct	Mean	SD	1	2	3	4	5	6
1. Ethical leadership	4.20	0.56	**0.786**					
2. Ethical GenAI usage	3.96	0.55	0.46^∗∗∗^	**0.780**				
3. Creative patient service	3.95	0.62	0.39^∗∗∗^	0.41^∗∗^	**0.816**			
4. AI compatibility	4.13	0.55	−0.12^∗∗∗^	−0.03	−0.06	**0.811**		
5. Gender	1.64	0.48	0.06	0.09^∗∗^	0.10^∗∗^	0.07		
6. Age	38.64	6.43	−0.04	−0.05	−0.04	0.15^∗∗∗^	0.05	
7. Experience	3.96	1.21	0.00	0.01	−0.12^∗∗∗^	0.01	−0.02	−0.04

*Note:* Source: Prepared by the authors based on results from the analysis. The bold diagonal values represent the square roots of the average variance extracted (AVE).

^∗∗^ *p* < 0.01.

^∗∗∗^ *p* < 0.001.

Data analysis was conducted using path analysis in Mplus 8.3 [45]. Path analysis was appropriate for testing the moderated mediation model because it estimates the direct, indirect and conditional effects simultaneously within a single model. Mediation was tested by estimating the indirect effect of ethical leadership on creative patient service through ethical GenAI usage, and moderated mediation was tested by allowing AI compatibility to moderate the first‐stage path from ethical leadership to ethical GenAI usage. Ethical leadership and AI compatibility were mean‐centred prior to creating the interaction term. The significance of indirect and conditional indirect effects was assessed using 5000 bootstrap resamples with 95% percentile confidence intervals, with effects considered significant when the interval excluded zero. The analysis proceeded in two stages: assessment of the measurement model for reliability and validity, followed by path analysis for hypothesis testing.

### 3.6. Common Method Bias

Because predictor and outcome variables were obtained from different sources, common method bias was expected to be limited. This was further examined statistically. Harman’s single‐factor test, using principal axis factoring, extracted five factors with eigenvalues above one, with the first factor explaining 33.90% of the variance, below the 50% criterion. In addition, a full collinearity test returned variance inflation factors (VIFs) below 3.0 for all constructs. Together, these results indicate that common method variance did not bias the findings [39, 46].

### 3.7. Measurement Model

The measurement model was evaluated for reliability, convergent validity and discriminant validity to ensure the constructs were adequately measured. All scales demonstrated acceptable reliability, with Cronbach’s alpha values ranging from 0.843 to 0.931 (exceeding the 0.70 threshold) and composite reliability (CR) values from 0.884 to 0.942 (exceeding 0.70) [47]. Convergent validity was supported by factor loadings generally above 0.70 (with minor exceptions still above 0.60, deemed acceptable in exploratory contexts) and average variance extracted (AVE) values from 0.609 to 0.666 (exceeding 0.50) [48]. Table 2 presents the item loadings, AVE, CR and Cronbach’s alpha for each construct.

Discriminant validity was confirmed using the Fornell–Larcker criterion. As shown in Table 3, the bolded diagonal values representing the square root of each construct’s AVE exceed the correlations with other constructs, with latent variable correlations ranging from −0.031 to 0.461 [49]. No collinearity issues were identified, with VIFs below 3.0 for all indicators and inner model paths. Overall, the measurement model demonstrated strong psychometric properties, allowing progression to structural model assessment.

### 3.8. Hypotheses Testing

The hypothesis testing was tested with 5000 bootstrap resamples and 95% percentile confidence intervals. AI compatibility and ethical leadership were mean‐centred prior to analysis. The models explained a moderate share of variance in the outcomes. Ethical leadership accounted for 21.3% of the variance in ethical GenAI usage (*R* ^2^ = 0.213, *F* (1, 590) = 159.64, *p* < 0.001), and this rose to 22.4% once AI compatibility and the interaction term were included (*R* ^2^ = 0.224, *F* (3, 588) = 56.49, *p* < 0.001). Together, ethical leadership and ethical GenAI usage explained 21.6% of the variance in creative patient service (*R* ^2^ = 0.216, *F* (2, 589) = 80.89, *p* < 0.001).

Hypotheses were tested via path coefficients, indirect effects and conditional effects (Table 4). Hypothesis 1 was supported, with ethical leadership positively related to creative patient service (*B* = 0.28, *p* < 0.001, 95% CI [0.190, 0.368]). Hypothesis 2 was supported, as ethical GenAI usage partially mediated the ethical leadership‐creative patient service relationship; the indirect effect was significant (*B* = 0.15, 95% CI [0.091, 0.206]), and the direct effect remained significant (*B* = 0.28, *p* < 0.001), indicating partial mediation. Hypothesis 3 was supported, with AI compatibility moderating the ethical leadership–ethical GenAI usage path (interaction *B* = 0.17, *p* < 0.01, 95% CI [0.051, 0.296]), such that the relationship was stronger at higher AI compatibility (+1 SD: *B* = 0.53, 95% CI [0.440, 0.617]) than at lower AI compatibility (−1 SD: *B* = 0.34, 95% CI [0.229, 0.443]). Hypothesis 4 was supported, as the conditional indirect effect of ethical leadership on creative patient service through ethical GenAI usage was stronger at higher AI compatibility (*B* = 0.18, 95% CI [0.107, 0.242]) than at lower AI compatibility (*B* = 0.11, 95% CI [0.062, 0.165]). The index of moderated mediation was significant (index = 0.06, 95% CI [0.014, 0.106]), confirming that the strength of the indirect effect depended on AI compatibility.

**TABLE 4:** Hypotheses testing.

Path/effect	B	SE	95% CI [LL, UL]
Total effect			
EL ⟶ CPS	0.43^∗∗∗^	0.04	[0.344, 0.509]
Direct effects			
EL ⟶ CPS	0.28^∗∗∗^	0.05	[0.190, 0.368]
EL ⟶ EGU	0.45^∗∗∗^	0.04	[0.379, 0.518]
EGU ⟶ CPS	0.33^∗∗∗^	0.05	[0.237, 0.421]
AIC ⟶ EGU	0.01	0.04	[‐0.063, 0.079]
Indirect effects			
EL ⟶ EGU ⟶ CPS	0.15	0.03	[0.091, 0.206]
Conditional direct effects			
EL × AIC ⟶ EGU (interaction)	0.17^∗∗^	0.06	[0.051, 0.296]
EL ⟶ EGU (at high AIC, +1 SD)	0.53^∗∗∗^	0.05	[0.440, 0.617]
EL ⟶ EGU (at low AIC, −1 SD)	0.34^∗∗∗^	0.05	[0.229, 0.443]
Conditional indirect effects			
EL ⟶ EGU ⟶ CPS (at high AIC, +1 SD)	0.18	0.04	[0.107, 0.242]
EL ⟶ EGU ⟶ CPS (at low AIC, −1 SD)	0.11	0.03	[0.062, 0.165]
Index of moderated mediation	0.06	0.02	[0.014, 0.106]

*Note: N* = 592. AIC = perceived AI compatibility. For indirect and conditional effects, SE is the bootstrap SE and CIs are 95% percentile bootstrap intervals (5000 resamples); these effects are significant when the interval excludes zero. All intervals shown exclude zero. AIC and EL were mean‐centred prior to analysis. Source: Prepared by the authors based on results from the analysis.

Abbreviations: CPS, creative patient service; EGU, ethical GenAI usage; EL, ethical leadership.

^∗∗^ *p* < 0.01.

^∗∗∗^ *p* < 0.001.

## 4. Discussion

This multi‐source study advances the understanding of how ethical leadership from head nurses shape nurses’ creative patient service in high‐pressure Chinese tertiary hospital settings, through the mediating role of ethical GenAI usage and moderated by perceived AI compatibility. Drawing on COR theory, the findings reveal that ethical leadership directly enhances creative patient service and indirectly via ethical GenAI usage, with the indirect effect strengthened at higher levels of AI compatibility. These results highlight the socio‐technical nature of GenAI integration in nursing, where leadership not only models ethical behaviours but also facilitates resource gain spirals that enable innovative, patient‐centred care amid workload pressures and staffing shortages [50]. By highlighting ethical GenAI usage as a key mechanism, the study highlights nursing leaders’ role in overcoming rational resistance, promoting AI literacy, and ensuring ethical scrutiny in AI‐driven workflows.

The finding that head nurses’ ethical leadership is positively related to nurses’ creative patient service is consistent with prior work linking ethical leadership to employee creativity and innovation in healthcare. Studies in nursing management have similarly reported that ethical leaders raise job satisfaction and innovative behaviour by encouraging value‐aligned conduct and reducing ethical dilemmas [18]. Our results extend this evidence from general innovation outcomes to creative patient service specifically and from Western and cross‐industry samples to the high‐pressure context of Chinese tertiary hospitals.

The mediating role of ethical GenAI usage aligns with research showing that responsible technology use transmits leadership effects into performance and creative outcomes [23–25]. Consistent with this work, we found that ethical leadership operates partly through the way nurses engage with GenAI, rather than on creative service alone. A point of divergence is that our mediation was partial rather than full: ethical leadership retained a direct effect on creative patient service alongside the indirect path. This suggests that ethical leadership shapes creative service through additional routes beyond GenAI use, such as psychological safety or intrinsic motivation, that were not modelled here. Studies reporting full mediation may reflect settings where the technology is more central to the task than it currently is in bedside nursing.

The moderating role of AI compatibility is directionally consistent with technology‐adoption research showing that perceived fit strengthens the influence of leadership and organisational factors on technology‐related behaviours [27, 28]. However, the interaction in our data was statistically significant but small. This weaker‐than‐expected effect may reflect the early stage of GenAI adoption in Chinese tertiary hospitals, where compatibility perceptions are still forming and may not yet differentiate strongly between nurses, or a restricted range in compatibility scores given the relatively high mean. Prior studies reporting stronger compatibility effects were often conducted with more mature technologies, where users hold more settled and varied perceptions of fit. This contrast points to compatibility as a boundary condition whose influence may grow as GenAI becomes embedded in routine nursing workflows.

### 4.1. Theoretical Implications

Theoretically, this research extends COR theory to the emerging context of GenAI adoption in nursing, demonstrating how ethical leadership acts as a vital external resource that mitigates loss spirals (e.g., ethical dilemmas or autonomy erosion) and initiates gain spirals through responsible technology use [22]. The partial mediation of ethical GenAI usage aligns with COR’s resource investment principle, showing that nurses invest leadership‐derived resources (e.g., ethical confidence) into verified, transparent GenAI practices, yielding creative outcomes like improvised patient solutions. Moreover, the moderating role of AI compatibility introduces a socio‐technical boundary condition, illustrating that alignment with workflows and values amplifies resource gains, while misalignment heightens threats, enriching COR’s application to complex, technology‐infused healthcare environments. This contributes to the recent call for bridging COR with socio‐technical frameworks, offering insights into how adaptive leadership supports ethical AI engagement, algorithmic bias mitigation and interdisciplinary collaboration in nursing practice [51].

### 4.2. Practical Implications

Practically, the findings provide evidence‐based guidance for nursing leaders and hospital administrators to ethically integrate GenAI, particularly in resource‐constrained settings like Chinese tertiary hospitals facing high turnover and workloads [52]. Head nurses should prioritise ethical role modelling, such as discussing AI ethics and emphasising responsible usage, to promote ethical GenAI adoption and unlock creative patient service, potentially improving satisfaction and outcomes. To draw on the moderating effect of AI compatibility, organisations should implement targeted initiatives like compatibility assessments, workflow‐aligned training programmes and cultural audits to enhance GenAI fit [53], thereby strengthening leadership impacts. These strategies align with the recent calls for distributed leadership approaches that address workforce readiness, reduce resistance through education on AI limitations and support equitable, resilient AI systems [54].

These insights also have direct implications for nursing education. Nursing schools should revise their curricula to prepare graduates for ethically grounded work with GenAI. We recommend embedding a dedicated component on AI ethics and responsible use into undergraduate and postgraduate nursing programmes, covering patient‐data privacy, verification of AI‐generated outputs, transparency and the preservation of professional judgement. Practical training that builds familiarity and perceived compatibility with clinical AI tools, delivered through simulation and supervised practice, would help new nurses translate ethical principles into day‐to‐day use. Complementary modules on ethical leadership for nurses moving into head nurse and management roles would extend these competencies to those who set the ethical tone for their units. Aligning nursing curricula with the realities of AI‐enabled practice in this way would support a workforce capable of using GenAI both responsibly and creatively.

### 4.3. Limitations and Future Research

Despite its strengths, this study has limitations. The cross‐sectional, dyadic design from 12 Chinese tertiary hospitals limits causal inferences and generalisability to other cultural or healthcare contexts, such as primary care or Western settings. Reliance on self‐reported measures may introduce common method bias [46], though the multi‐source design mitigated this. Furthermore, the emerging nature of GenAI tools means the scales, while adapted, require further validation in diverse samples. Future research should adopt longitudinal designs to track resource dynamics over time and explore objective outcomes like patient satisfaction metrics. Expanding to include other moderators (e.g., organisational culture or algorithmic bias perceptions) or mediators (e.g., AI literacy) could deepen socio‐technical insights. Comparative studies across global healthcare systems would inform interdisciplinary and cross‐cultural leadership strategies for ethical AI integration.

## 5. Conclusion

This multi‐source study of 592 staff nurses and 64 head nurses across 12 Chinese tertiary hospitals examined how head nurses’ ethical leadership shapes nurses’ creative patient service in the era of generative AI. Three findings stand out. First, ethical leadership was positively related to creative patient service. Second, this relationship was partially explained by nurses’ ethical GenAI usage, indicating that ethical leaders support creative care in part by guiding how nurses engage with GenAI tools. Third, perceived AI compatibility strengthened the link between ethical leadership and ethical GenAI usage, so the indirect effect on creative patient service was greater among nurses who perceived GenAI as fitting their workflows and values. Drawing on COR theory, these results position ethical leadership as an external resource that helps nurses invest in responsible GenAI use and convert it into creative patient care. For nursing management, the findings point to ethical role modelling by head nurses, deliberate attention to AI compatibility and curriculum reform in nursing education as complementary levers for the responsible and innovative integration of GenAI. As GenAI becomes embedded in clinical practice, ethical leadership at the unit level will be central to ensuring that technological capability translates into better and more creative patient service.

## Funding

No funding was received for this manuscript.

## Conflicts of Interest

The authors declare no conflicts of interest.

## Data Availability

The data that support the findings of this study are available from the corresponding author upon reasonable request.
